# Comparative gene expression study highlights molecular similarities between triple negative breast cancer tumours and feline mammary carcinomas

**DOI:** 10.1111/vco.12800

**Published:** 2022-01-14

**Authors:** Lara Sommerville, Jane Howard, Shane Evans, Pamela Kelly, Amanda McCann

**Affiliations:** ^1^ UCD Conway Institute of Biomolecular and Biomedical Research University College Dublin Dublin Ireland; ^2^ UCD School of Medicine, College of Health and Agricultural Sciences (CHAS) University College Dublin Dublin Ireland; ^3^ UCD School of Veterinary Medicine, Veterinary Sciences Centre University College Dublin Dublin Ireland

**Keywords:** comparative oncology, feline mammary carcinoma, genome, molecular biology, triple negative breast cancer

## Abstract

Triple negative breast cancer (TNBC) is a rare, highly metastatic subtype of breast cancer that typically develops tumours of a high histological grade. As TNBC is negative for the oestrogen, progesterone and HER2 receptors it is also not eligible for targeted hormonal therapies. Therefore, those diagnosed with TNBC are faced with a very poor prognosis. Feline mammary carcinomas (FMCs) have been shown to share key characteristics of TNBC and are being investigated as novel animal models of this disease. A study by Granados‐Soler et al., investigating prognostic markers of FMCs provided the basis of this research, and their prognostic value in TNBC was evaluated using a ‘*data‐mining*’ research approach. Overall, the comparative genomic aspect of this research identified several potential prognostic markers translatable across TNBC and FMCs. These prognostic markers warrant further investigation in comparative oncology studies.

Comparative oncology, which involves integrating naturally occurring cancers in companion animals into studies focusing on human cancer, holds promise in accelerating cancer drug discovery and development. This research approach offers many advantages that overcome the main limitations associated with traditional rodent models. These include the failure of rodents to accurately replicate the biological properties and genetic diversity of human cancer, as well as predicting the effects of therapeutic changes and tumour‐immune system interactions in human subjects. In comparison, spontaneously occurring tumours in animals develop naturally over a long period of time, grow in the presence of an intact immune system and share histological features with tumours of their human counterpart. These companion animals also share the same external environment as humans and are therefore exposed to the same environmental toxins and carcinogens. In practice, comparative oncology studies have mostly focused on canine cancer, with the National Cancer Institute's Comparative Oncology Trial Consortium consisting of 22 research sites across North America that focus on using canine cancer to aid in drug development.^1^ Similarly, the Canine Comparative Oncology and Genomics Consortium received $1.1 million in funding from Pfizer which facilitated the completion of a biospecimen repository containing over 60 000 samples from 2000 dogs.^2^ While naturally occurring canine tumours are positively impacting comparative research, the relevance of the domestic cat in comparative studies is gradually emerging.^3^ This article will focus on mammary tumours of the feline species, which are showing promise as models of human triple negative breast cancer (TNBC) in particular.

TNBC is a rare, highly metastatic subtype of breast cancer that typically develops tumours of a high histological grade. As it is not eligible for targeted hormonal therapies, those diagnosed with TNBC are offered limited treatment options. Overall, this leads to a poor prognosis and lower 5‐year survival rate than other forms of breast cancer.^4^ Studies have shown that a high proportion of feline mammary carcinomas (FMCs) can be classified according to the triple negative subtype and similarly exhibit highly aggressive behaviour.^5^, ^6^ Furthermore, FMCs display the ‘*basal‐like*’ TNBC subtype, which is defined as having increased expression levels of P‐cadherin, epidermal growth factor receptor and cytokeratins 5/6, 14 and 17.^7^, ^8^ The ‘*basal‐like*’ subtype is clinically significant in humans as it typically presents in younger women and is associated with shortened survival intervals.^9^, ^10^ Molecular comparisons of FMCs and TNBCs may further support the use of FMCs as an appropriate model to study TNBC.

In a recent article titled, ‘Analysis of Copy Number Variations and Feline Mammary Carcinoma Survival’,^11^ Granados‐Soler et al., analysed the association between cancer‐related genes and survival outcomes in FMCs. Notably, copy number variations (CNVs), defined as copy number losses (CNLs) or copy number gains (CNGs), were investigated in great detail in '*basal‐like'* triple‐negative (BL‐TN) FMC samples. The malignant grade of the FMC samples in this study were classified according to the Elston and Ellis and FMC‐adapted Mills grading system,^12^ and FMCs were further divided into molecular subtypes based on the human breast cancer St. Gallen molecular classification. In total, 13/33 (39%) tumours were classified as triple negative which could be further divided into basal‐like (5/13) and normal‐like tumours (8/13), respectively. Similar to human breast cancer, each subtype of FMC correlated to differences in prognosis. Specifically, the BL‐TN FMCs had the highest frequency of CNVs and displayed the worst prognosis over a follow‐up period of 2 years (disease free survival [DFS], *p* < .001; OS, *p* < .00001). These CNVs included CNGs in protein serine/threonine kinases, like MAP2K8 and PRKCQ, that are involved in cellular proliferation and motility. Alternatively, CNLs that were common to BL‐TN FMCs were found to be enriched in the basal‐cell carcinoma and Wnt signalling pathways, which are typically dysregulated in human TNBC. The BL‐TN FMC subtype also displayed additional CNLs compared to the other molecular subtypes, which were associated with reduced DFS and found in the chromosomal regions A1 105–124 Mb and D2 54–88.9 Mb.

DFS is described by the National Cancer Institute (NCI) as the length of time after primary treatment for a cancer finishes that the patient survives without any signs or symptoms of that cancer.^13^ Oncomine^14^ is a cancer array database and data‐mining platform, aimed at facilitating cancer gene discovery from genome‐wide expression analysis. Oncomine contains 65 gene expression datasets made up of nearly 48 million gene expression measurements from over 4700 microarray experiments. This database compares differential expression analysis in normal tissue and most types of cancer by measuring DNA copy number and mRNA expression levels.^14^ Additionally, clinical, and pathological data are also available. This powerful bioinformatic platform therefore allows users to analyse publicly available gene expression data for specific or multiple genes in a selected cancer analysis. BreastMark is a breast cancer specific web‐based system which allows the investigation of genes significantly associated with survival in breast cancer.^15^ The algorithm combines gene expression data from multiple microarray experiments and correlates the expression levels to detailed clinical data. Twenty‐six datasets on 12 microarray platforms, corresponding to ~17 000 genes are integrated into analysis by BreastMark.^15^ This tool allows the preliminary, bioinformatic assessment of potential biomarkers in breast cancer.

Using 14 of the markers outlined by Granados‐Soler et al., we analysed for expression and impact on DFS in TNBC using the Oncomine^14^ and BreastMark^15^ publicly available databases. These markers were chosen for comparison since they were (i) common to BL‐TN FMC tumours and (ii) associated with reduced DFS. Specifically, SYK and JAK2 were analysed for incidence of CNGs whilst WNT5A, MAGI1, MITF, SUCLG2, PDHB, NTM, BARX2, CDON, FEZ1, CD44, FZD4 and ME3 were analysed for incidence of CNLs.

Table 1 shows that increased expression of JAK2 and SYK were found to be significantly associated with favourable DFS in TNBC (SYK *p* = .05, HR = 0.5293; JAK2 *p* = .0102, HR = 0.4144). Although Oncomine suggests that SYK is significantly over‐expressed in TNBC (*p* = .002), it did not indicate SYK as a CNG in TNBC (*p* = .133). On the other hand, JAK2 was found to be significantly amplified in TNBC (*p* = 4.58 × 10^−9^). Conversely, increased expression of JAK2 associated with a more favourable prognosis according to BreastMark (*p* = .0102, HR = 0.4144). This deviated away from the results of Granados‐Soler et al., where it was suggested that amplification of JAK2 associated with reduced DFS (*p* = .009). In this case, the results of the BreastMark analysis do not accurately reflect TNBC, as the chromosomal region *9p24*, which contains JAK2, is known to be commonly amplified in TNBC and associated with poor survival outcomes.^16^ Interestingly, Granados‐Soler et al. found that the corresponding feline homologue region of D4 1–16.7 Mb was amplified in 5/11 triple‐negative FMC samples, strengthening the argument that FMCs are genetically similar to TNBC.

**TABLE 1:** Comparison of CNGs common to BL‐TN FMCs and associated with reduced DFS, to equivalent homologues in human TNBC

	FMC			TNBC			
Protein	BLAST alignment	CNG/CNL	DFS	Expression	CNG/CNL	DFS	HR
SYK	92.6%	CNG	*p* = .0009*	Over‐expressed* (*p* = .002)	CNG (*p* = .133)	*p* = .05*	0.5293
JAK2	94.5%	CNG	*p* = .009*	Over‐expressed (*p* = .14)	CNG* (*p* = 4.58 × 10^−9^)	*p* = .0102*	0.4144

*Note*: mRNA expression levels and incidence of CNG/CNL in TNBC is obtained from Oncomine and association of the marker with DFS is calculated through BreastMark. A hazard ratio (HR) ≥1 indicates that increased expression of the marker is associated with poor prognosis, whereas a HR ≤1 means that increased expression of the marker is associated with a good prognosis. The log‐rank *p* values determined by BreastMark correspond to differences in survival. In all analyses a *p* value <.05 is considered statistically significant.

Abbreviations: CNG, copy number gain; CNL, copy number loss; DFS, disease free survival; FMC, feline mammary carcinoma; TNBC, triple negative breast cancer.

* Significant *p* value.

Table 2 demonstrates that two CNLs identified by Granados‐Soler et al., MITF and SUCLG2, were also implicated as CNLs in TNBC. These markers also demonstrated significantly decreased expression (MITF, *p* = .045, SUCLG2, *p* = .01). BreastMark also indicated that SUCLG2 has an association with DFS trending towards significance, in which increased expression of SUCLG2 may correlate with a poor prognosis (*p* = .075, HR = 1.799). This disagrees with the findings of Granados‐Soler et al. where deletion of SUCLG2 was associated with reduced DFS (*p* = .009). The exact role of SUCLG2 in breast cancer is not understood. However, one study by Hart et al., found that targeting of SUCLG2 in mesothelial cells (that make up the ovarian cancer tumour microenvironment), lead to reduced cellular invasion in an organotypic 3D model.^17^ SUCLG2 has also been identified as a potential novel biomarker of follicular cancer.^18^ WNT5A, MAGI1, PDHB, BARX2, CD44 and ME3 did not yield any significant levels of over‐/under‐expression, incidence of copy number alterations or association with changes in DFS in TNBC.

**TABLE 2:** Comparison of CNLs common to BL‐TN FMCs and associated with reduced DFS, to equivalent human homologues in human TNBC

	FMC			TNBC			
Protein	BLAST alignment	CNG/CNL	DFS	Expression	CNG/CNL	DFS	HR
WNT5A	99.2%	CNL	*p* = .009*	Under‐expressed (*p* = .185)	CNL (*p* = .163)	*p* = .719	1.129
MAGI1	93.6%	CNL	*p* = .009*	Under‐expressed (*p* = .13)	CNL (*p* = .299)	*p* = .233	0.6578
MITF	98%	CNL	*p* = .009*	Under‐expressed* (.045)	CNL* (*p* = .029)	*p* = .612	1.184
SUCLG2	86.323%	CNL	*p* = .009*	Under‐expressed* (.01)	CNL* (*p* = .007)	*p* = .075**	1.799
PDHB	95%	CNL	*p* = .009*	Under‐expressed (.221)	CNL (*p* = .116)	*p* = .639	1.174
NTM	95.8%	CNL	*p* < .0001*	Under‐expressed* (.007)	CNL (*p* = .891)	*p* = .429	1.304
BARX2	91.4%	CNL	*p* < .0001*	Under‐expressed (.855)	(−)	*p* = .164	0.6141
CDON	83.6%	CNL	*p* < .0001*	Under‐expressed** (.061)	CNL (*p* = .988)	*p* = .0414*	0.4504
FEZ1	97.7%	CNL	*p* < .0001*	Under‐expressed** (.074)	CNL (*p* = .984)	*p* = .93	0.9709
CD44	68.9%	CNL	*p* = .004*	Under‐expressed (.94)	CNL (*p* = .933)	*p* = .233	0.6676
FZD4	97.8%	CNL	*p* = .004*	Under‐expressed** (.055)	CNL (*p* = 1)	*p* = .7388	0.8924
ME3	97.5%	CNL	*p* = .004*	Under‐expressed (.103)	(−)	*p* = .528	0.7959

*Note*: mRNA expression levels and incidence of CNG/CNL in TNBC is obtained from Oncomine and association of the marker with DFS is calculated through BreastMark. A hazard ratio (HR) ≥1 indicates that increased expression of the marker is associated with poor prognosis, whereas a HR ≤1 means that increased expression of the marker is associated with a good prognosis. The log‐rank *p* values determined by BreastMark correspond to differences in survival. In all analyses a *p* value <.05 is considered statistically significant.

Abbreviations: CNG, copy number gain; CNL, copy number loss; DFS, disease free survival; FMC, feline mammary carcinoma; TNBC, triple negative breast cancer.

* Significant *p* value.

** Trending towards significance (−) no data available.

Overall, comparing the gene expression of TNBCs and FMCs identified several potential prognostic markers which are translatable across both species. These markers could be used in further comparative oncology studies investigating TNBC pathogenesis and novel therapeutic targets. Further comparative genome studies between TNBC and FMC cohorts, such as that conducted by Granados‐Soler et al., are needed to validate the use of FMCs as comparative animal models of TNBC. Importantly, many of the markers investigated in this study demonstrated high levels of homology between the feline and human genomes, as demonstrated by the BLAST alignments in Tables 1 and 2. In fact, 11/14 markers included in this study demonstrated >90% identity to their human homologue. This emphasizes the high degree of similarity between both species and ultimately supports the argument that FMCs are valuable resources in terms of TNBC research.

## Data Availability

Data sharing not applicable to this article as no datasets were generated or analysed during the current study.
